# Clinical Symptoms, Laboratory Parameters and Long-Term Follow-up in a National DADA2 Cohort

**DOI:** 10.1007/s10875-023-01521-8

**Published:** 2023-06-05

**Authors:** Marie Valérie E. Andriessen, G. Elizabeth Legger, Robbert G. M. Bredius, Marielle E. van Gijn, A. Elisabeth Hak, Petra C. E. Hissink Muller, Sylvia Kamphuis, Femke C. C. Klouwer, Taco W. Kuijpers, Helen L. Leavis, Stefan Nierkens, Abraham Rutgers, Lars T. van der Veken, Gijs T. J. van Well, Catharina M. Mulders-Manders, Joris M. van Montfrans

**Affiliations:** 1 Department of Pediatric Immunology and Infectious Diseases, University Medical Center Utrecht, Utrecht University, PO Box 85050, 3508 GA Utrecht, the Netherlands; 2 Department of Pediatric Rheumatology, University of Groningen, University Medical Center Groningen, Groningen, the Netherlands; 3 Department of Pediatrics, Willem-Alexander Children’s Hospital, Leiden University Medical Center, Leiden, the Netherlands; 4 Department of Genetics, University of Groningen, University Medical Center Groningen, Groningen, the Netherlands; 5 Departments of Internal Medicine and Rheumatology and Clinical Immunology, Amsterdam UMC, Amsterdam, the Netherlands; 6 Department of Pediatric Rheumatology, Sophia Children’s Hospital, Erasmus MC University Centre, Rotterdam, the Netherlands; 7 Department of Neurology and Pediatric Neurology, Location AMC, Amsterdam University Medical Centers, University of Amsterdam, Amsterdam, the Netherlands; 8 Department of Pediatric Immunology, Rheumatology and Infectious Diseases, Emma Children’s Hospital, Amsterdam University Medical Centers, University of Amsterdam, Amsterdam, the Netherlands; 9 Department of Rheumatology & Clinical Immunology, University Medical Center Utrecht, Utrecht University, Utrecht, the Netherlands; 10 Center for Translational Immunology, University Medical Center Utrecht & Princess Máxima Center for Pediatric Oncology, Utrecht, the Netherlands; 11 Department of Rheumatology and Clinical Immunology, University of Groningen, University Medical Center Groningen, Groningen, the Netherlands; 12 Department of Genetics, Division Laboratories, Pharmacy and Biomedical Genetics, University Medical Center Utrecht, Utrecht University, Utrecht, the Netherlands; 13 Department of Pediatrics: Division of Pediatric Infectious Diseases, Immunology and Rheumatology, Maastricht University Medical Center, Maastricht, the Netherlands; 14 Department of Internal Medicine, Radboud Expertise Center for Immunodeficiency and Autoinflammation, Radboud University Medical Center, Nijmegen, the Netherlands

**Keywords:** DADA2, follow-up, treatment, complications, HLH

## Abstract

Deficiency of adenosine deaminase-2 (DADA2) is an autosomal recessive autoinflammatory disease with an extremely variable disease presentation. This paper provides a comprehensive overview of the Dutch DADA2 cohort. We performed a retrospective cohort study in 29 ADA2-deficient patients from 23 families with a median age at inclusion of 26 years. All patients had biallelic pathogenic variants in the *ADA2* gene. The most common clinical findings included cutaneous involvement (79.3%), (hepato)splenomegaly (70.8%) and recurrent infections (58.6%). Stroke was observed in 41.4% of the patients. The main laboratory abnormalities were hypogammaglobulinemia and various cytopenias. Patients presented most often with a mixed phenotype involving vasculopathy, immunodeficiency and hematologic manifestations (62.1%). In this cohort, malignancies were reported in eight patients (27.6%), of whom five presented with a hematologic malignancy and two with a basal cell carcinoma. Four patients developed hemophagocytic lymphohistiocytosis (HLH) or an HLH-like episode, of whom three passed away during or shortly after the occurrence of HLH. TNF-inhibitors (TNFi) were effective in treating vasculopathy-associated symptoms and preventing stroke, but were hardly effective in the treatment of hematologic manifestations. Three patients underwent hematopoietic cell transplantation and two of them are doing well with complete resolution of DADA2-related symptoms. The overall mortality in this cohort was 17.2%. In conclusion, this cohort describes the clinical, genetic and laboratory findings of 29 Dutch DADA2 patients. We describe the occurrence of HLH as a life-threatening disease complication and report a relatively high incidence of malignancies and mortality.

**Supplementary Information:**

The online version contains supplementary material available at 10.1007/s10875-023-01521-8.

## Introduction

Deficiency of adenosine deaminase-2 (DADA2) was first described in 2014 as an autosomal recessive autoinflammatory disease caused by loss of function variants in the *ADA2* gene [1, 2]. Initially, DADA2 was characterized as a vasculopathy with either early onset stroke or cutaneous vasculitis as main presenting symptom [1, 2]. This clinical phenotype has been expanded by demonstrating that at least 50% of patients exhibit hematologic or immunologic symptoms, often without signs of vasculopathy [3–5]. DADA2 manifestations are frequently divided into three phenotypes: inflammatory vasculopathy, immunodeficiency and hematologic manifestations [4, 6]. Recently, Lee and colleagues described a genotype–phenotype association, in which missense pathogenic variants with residual ADA2 activity were associated with a vasculopathy phenotype, whereas more detrimental missense variants, indels, and nonsense variants leading to complete loss of ADA2 function were more often associated with hematologic manifestations [6]. However, the genotype–phenotype relation is still debated, as certain pathogenic variants were associated with a single phenotype, while others were seen in multiple phenotypes and even family members with identical pathogenic variants may exhibit variable phenotypes [6–9].

Although the exact pathophysiology of DADA2 is unclear, a zebrafish model suggests that ADA2 is important for vascular integrity and development of hematopoietic cells [1]. ADA2 is an extracellular enzyme primarily expressed by monocytes and macrophages [4, 10]. Absence of ADA2 results in skewing of macrophages to a pro-inflammatory M1 phenotype, chronic neutrophil activation and increased numbers of nonclassical monocytes [1, 10, 11]. Neutrophils and low density granulocytes (LDG’s) were found to express enhanced neutrophil extracellular trap (NET) formation in the absence of ADA2 [12]. These mechanisms lead to increased production of pro-inflammatory cytokines, such as tumor necrosis factor (TNF)-alpha and interferon-gamma (INF-γ) by nonclassical monocytes and M1 macrophages [13]. TNF-inhibitors (TNFi) are the cornerstone of DADA2 treatment. TNFi effectively prevent strokes, and are effective against febrile episodes and vasculopathy [3, 6, 14–16]. Meanwhile, TNFi were reported to be less effective for immunodeficiency and hematologic manifestations [6, 17]. Patients at risk for infections benefit from antibiotic prophylaxis and/or immunoglobulin replacement therapy [2, 3, 7, 8, 15]. For patients with severe hematologic or immunologic manifestations, hematopoietic cell transplantation (HCT) has successfully been performed, resulting in full resolution of the vascular, immunologic and hematologic phenotype of DADA2 [3, 15, 18, 19]. Therefore, HCT is particularly indicated in patients with refractory immunodeficiency or severe hematologic disease [3, 4].

The variability in clinical disease course, even in patients with the same genotypic variants in the *ADA2* gene, precludes uniform treatment of all DADA2 patients. Currently, it is recommended to offer TNFi to all symptomatic DADA2 patients, even those who are only mildly symptomatic, because of the increased stroke risk. To date, it is unclear if there is a subgroup of patients without an increased risk for stroke who would not require livelong TNFi. It is also unknown whether a subset of patients would benefit from pre-emptive HCT, prior to development of severe hematologic manifestations such as bone marrow failure, malignancy or hemophagocytic lymphohistiocytosis (HLH).

This paper aims to contribute to the existing body of knowledge by providing a comprehensive overview of clinical, laboratory and genetic characteristics of the Dutch DADA2 cohort. We focus on genotype and phenotype of DADA2 patients and report on rare manifestations including malignancies and HLH. We describe the response to TNFi for patients with different phenotypes and assess the relation between ADA2 residual activity and phenotype.

## Methods

### Design

Retrospective follow-up study.

### Patients

Patients were identified by contacting all physicians of the immunology departments of the seven Dutch university hospitals, and by searching the Eurofever Registry and the Dutch national immunodeficiency database. Eurofever is an ongoing international registry for autoinflammatory diseases initiated by the Pediatric Rheumatology International Trials Organisation (PRINTO) [20]. The Dutch national immunodeficiency database is an ongoing prospective study on immunodeficiencies approved by the Medical Ethical Board of the Erasmus MC in Rotterdam, the Netherlands (National PID study, METC: NL40331.078). Informed consent was obtained from all the included patients. Eleven patients were described previously (patients 1–5, 8, 14, 16, 20, 21, 23) [8, 21]. We studied the complete disease history per patient, starting from suspected first symptoms until the latest clinical contact. Follow-up varied widely between 2 months and more than 10 years.

### Clinical Data Collection

A predefined clinical data set on disease course, laboratory values, imaging, genetic evaluations, treatment and treatment outcome was collected from the electronic patient files and the treating physicians. This data set was compiled on the basis of previous literature.

### ADA2 Enzyme Activity

ADA2 enzyme activity was measured as previously described [8]. Results reported in clinical practice were used. No new blood samples were taken for this study.

### Genotype

All patients demonstrated biallelic pathogenic variants in the *ADA2* gene. These pathogenic variants were collected and correlated to the predominant phenotypes.

### Phenotype

To categorize the patients in this study by ‘predominant phenotype’, we used the definitions of vasculopathy, immunodeficiency and hematologic manifestations, or a combination of these (‘mixed phenotype’), as described by Lee et al. [4]. We assessed all symptoms during the patient’s disease course. Laboratory values, measured during routine medical care, were obtained retrospectively at representative time points in the disease course. For thrombocytopenia, neutropenia, leukopenia, lymphopenia, anemia and hypogammaglobulinemia, age-specific reference values were used.

### Statistics

Skewed data were presented as medians with interquartile ranges (IQR). Mann–Whitney *U* tests were performed to compare median ADA2 enzyme activity between patients with and without stroke and between patients with a predominant hematologic and a predominant vasculopathy phenotype. For these analyses, we only used residual ADA2 enzyme activity values measured before initiation of TNFi. Categorical variables were compared using the Chi-square test or the Fisher exact test depending on the sample size. IBM SPSS Statistics 21 was used for data analysis.

## Results

### Patients

We identified 32 DADA2 patients in the Netherlands, of whom 29 patients from 23 families provided informed consent and were included in the study.

### Clinical Presentation

Baseline characteristics are summarized in Table 1 and Supplementary Table 1. Median age at disease presentation was 5 years (IQR 14 years). Median age at inclusion was 26 years (IQR 31.5 years).

**Table 1:** Baseline characteristics of DADA2 patients (*n* = 29)

	Number (Interquartile range (IQR)/percentage)
Median age at inclusion (years)	26 (IQR 31.5)
Median age at disease onset (years)	5 (IQR 14)
Male gender	17 (58.6%)
Alive	24 (82.8%)
ADA2 genotype	
c.506G > A p.(Arg169Gln) homozygous	10 (34.5%)
c.973-2A > G p.(?) homozygous	4 (13.8%)
Other	15 (51.7%)
Symptoms	
Cutaneous involvement	23 (79.3%)
Fever	12 (48.0%)
Stroke	12 (41.4%)
PAN-like rash or other cutaneous vasculitis	10 (34.5%)
Systemic vasculitis	1 (3.4%)
Recurrent infections	17 (58.6%)
Arthralgia/artritis	9 (31.0%)
IBD-like symptoms	2 (6.9%)
Aphthous stomatitis	11 (37.9%)
(Hepato)splenomegaly	17 (70.8%)
Malignancy (all types, including basal cell carcinoma)	8 (27.6%)
Laboratory values	
Anemia	14 (48.3%)
Thrombocytopenia	11 (37.9%)
Leukopenia	16 (55.2%)
Neutropenia	14 (48.3%)
Lymphopenia	9 (32.1%)
Hypogammaglobulinemia	14 (50.0%)
Hemophagocytic lymphohistiocytosis (HLH)	4 (13,8%)
Predominant phenotype	
Inflammatory vasculopathy phenotype	7 (24.1%)
Hematologic phenotype	4 (13.8%)
Mixed phenotype*	18 (62.1%)

PAN, polyarteritis nodosa; IBD, inflammatory bowel disease

^*^Mixed phenotype indicates a combination of two or three phenotypes (vasculopathy, hematologic manifestations, immunodeficiency)

The most common clinical findings included cutaneous involvement (79.3%), (hepato)splenomegaly (70.8%), recurrent infections (58.6%), recurrent fever (48.0%), stroke (41.4%) and vasculitis (PAN-like skin disease or other types of cutaneous vasculitis in 34.5%; systemic vasculitis in 3.4%; Table 1 and Supplementary Table 1). Table 2 shows the clinical presentation and predominant phenotype of all the included patients. Patients presented most often with a mixed phenotype (62.1%), followed by a predominant vasculopathy phenotype (24.1%) and a predominant hematologic phenotype (13.8%). None of our patients exhibited a pure immunodeficiency phenotype (i.e. without hematologic abnormalities or vasculopathy, Table 2).

**Table 2:**
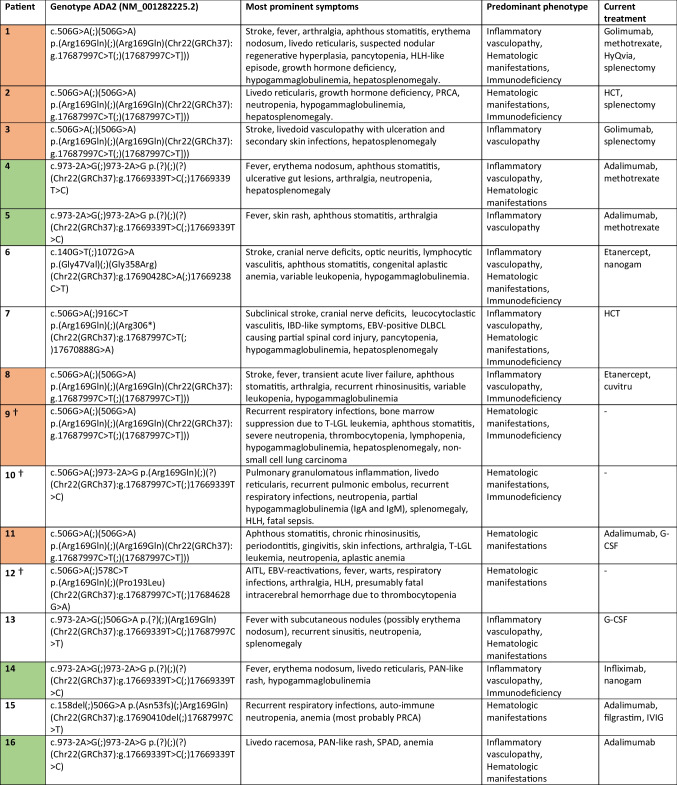

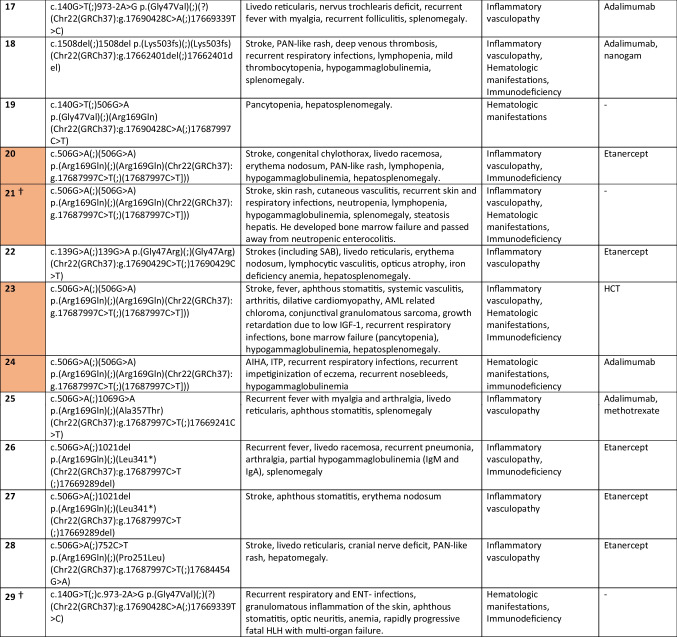
Genotype, most prominent symptoms, predominant phenotype and treatment (*n* = 29)

X= homozygous c.506G>A p.(Arg169Gln) pathogenic variant , X= homozygous c.973-2A>G p.(?) pathogenic variant. Abbreviations; HLH= Hemophagocytic lymphohistiocytosis; PRCA= Pure red cell aplasia; HCT= Hematopoietic cell transplantation; IBD-like= Inflammatory bowel disease like; EBV-positive DLBCL= Epstein-Barr virus positive diffuse large B-cell lymphoma; T-LGL= T-cell large granular lymphocytic; AITL= Angio-immunoblastic T-cell lymphoma; PAN= polyarteritis nodosa; SPAD= Specific polysaccharide antibody deficiency; SAB= Subarachnoid hemorrhage; AML= Acute myeloid leukemia; IGF-1= Insulin-like growth factor 1; AIHA= Auto-immune hemolytic anemia; ITP= Immune thrombocytopenic purpura, ENT= Ear nose throat; TNF= Tumor necrosis factor; IVIG= Intravenous immunoglobulins

Patients with low CD19+ B-cells without hypogammaglobulinemia are not considered immunodeficiency phenotype. Patients with solely decreased IgM or IgA are not counted as hypogammaglobulinemia

### Cytopenias and Hypogammaglobulinemia

Laboratory values of all patients are shown in the Supplementary Table 2A–B. A large proportion of patients developed transient or persistent anemia (48.3%), leukopenia (55.2%) or neutropenia (48.3%) during their disease course (Table 1). Two patients were diagnosed with pure red cell aplasia (PRCA; Table 2). Thrombocytopenia was seen in 37.9% of patients and lymphopenia in 32.1%. Altogether, 21/29 patients (72.4%) had transient or persistent cytopenia in one or multiple cell lines. Of them, 13 patients had splenomegaly (61.9%). Hypogammaglobulinemia was seen in 50.0% of the patients.

### Neurological Symptoms

Twelve patients (41.4%) encountered one or more episodes of ischemic stroke. One of them also developed an intracerebral hemorrhage and one a subarachnoid hemorrhage. In addition to these twelve patients, patient 12 presumably encountered (fatal) intracerebral hemorrhage associated with severe thrombocytopenia; however, this could not be proven as no CT-scan was performed. Clinical manifestations of patients with and without stroke were compared in Table 3. Patient 12 was excluded from this table. PAN-like skin disease or other types of vasculitis (including cutaneous and systemic vasculitis) was seen in 9 of the 12 patients with stroke, compared to 2 of the 16 patients without stroke (*P* = 0.001). Other clinical manifestations did not differ significantly between patients with and without stroke (Table 3). In this cohort, stroke was seen as presenting a symptom of DADA2 in several patients, whereas in other patients the stroke(s) occurred later in their disease courses. The retrospective nature of this study precluded a precise quantification of the number of patients with stroke as presenting symptom, as we could not reliably assess the presence of pre-stroke DADA2-related symptoms in all patients.

**Table 3:** Clinical manifestations in patients with stroke and without stroke

	Stroke (*n* = 12)	No stroke (*n* = 16)	*p*-value
Cutaneous involvement	10	12	0.67
Fever	3	8	0.12
PAN-like rash or other vasculitis (including cutaneous and systemic vasculitis)	9	2	**0.001**
Arthralgia/artritis	3	5	1.00
IBD-like disease	1	1	1.00
Aphthous stomatitis	5	6	1.00
Anemia	5	8	0.72
Thrombocytopenia	5	5	0.70
Neutropenia	4	9	0.28
Lymphopenia	5	3	0.40
Hypogammaglobulinemia	8	6	0.25

Patient 12 was excluded from this table because no CT-scan was performed to confirm intracerebral hemorrhage

Other neurological symptoms in this cohort varied in severity and included headache, seizures, optic neuritis, vertigo and cranial nerve deficits (causing hearing loss, vertigo, optic nerve atrophy, oculomotor disturbances due to third, fourth or sixth cranial nerve palsy). Some of these cranial nerve deficits were associated with stroke. Patient 2 developed severe headache and vomiting due to a jugular vein thrombosis associated with a central venous line and subsequently developed increased intracranial pressure and hydrocephalus, for which third ventriculostomy was performed. Patient 7 suffered from partial spinal cord injury and Horner’s syndrome due to an EBV-positive diffuse large B-cell lymphoma causing compression.

### Malignancies

In this cohort, 8 patients (27.6%) developed one or more malignancies, including five hematologic malignancies (Table 4). Two of these patients (25%) passed away. Malignancy was the presenting symptom in three patients (patient 7, 12 and 23). Patient 7 developed an EBV-positive diffuse large B-cell lymphoma, which came in remission with chemotherapy (Table 4). After genetic testing showed biallelic pathogenic variants in the *ADA2* gene, the patient developed pancytopenia and a perianal fistula probably due to inflammatory bowel disease (IBD) in the context of DADA2. Infliximab, ciclosporin and prednisone were initiated, resulting in improvement of anemia and lymphocyte-driven immune-mediated neutropenia and remission of the IBD-like disease. Eventually, the patient underwent HCT as described later. Patient 9 developed a T-cell large granular lymphocytic (T-LGL) leukemia and associated myelofibrosis, which failed to respond to several lines of T-LGL treatment and TNFi (Table 4). The patient was subsequently diagnosed with non-small cell lung carcinoma, which precluded referral for HCT and she eventually passed away in palliative care setting. Patient 11 was also diagnosed with a T-LGL leukemia which did not respond to T-LGL treatment regimens and TNFi (Table 4). HCT is currently under consideration as definitive treatment. Patient 12 presented with an angio-immunoblastic T-cell lymphoma (AITL), which initially responded to chemotherapy and autologous HCT (Table 4). During post-transplantation follow-up, the lymphoma presumably recurred and was complicated by HLH. The patient eventually passed away most likely as a result of thrombocytopenia-associated intracerebral hemorrhage. Patient 23 suffered from acute myeloid leukemia (AML) related chloroma for which she was treated according to the European AML-15 protocol and conjunctival granulomatous sarcoma. She underwent HCT and 6 years later, she developed a progressive schwannoma of the ischiadic nerve. Recently, 9 years post-HCT, she developed a myxoid liposarcoma in the upper leg for which treatment is being scheduled. Patient 17 and 20 developed basal cell carcinomas of the skin at relatively young age (18 and 26 years, respectively), which were successfully treated with surgical excision. Patient 3 was diagnosed with prostate carcinoma for which radical prostatectomy was performed. Of note, the majority of the patients with a malignancy had homozygous or (compound) heterozygous R169Q pathogenic variants.

**Table 4:** Overview of malignancies

Patient	Pathogenic variants	Age at genetic confirmation of DADA2 (years)	Age at developing malignancy (years)	Duration of TNFi treatment at onset of malignancy (years)	Phenotype (V/H/I/M)	Malignancy	Treatment	Outcome
3	R169Q/ R169Q	50	56	6	V	Prostate carcinoma	Radical prostatectomy	Radical resection of prostate carcinoma
7	R169Q/ R306*	29	28	0	M	EBV-positive diffuse large B-cell lymphoma	Surgical decompression, dexamethasone, chemotherapy (6 × R-CHP and 6 × methotrexate), rituximab monotherapy *After DADA2 was diagnosed*: infliximab/ciclosporin/prednisolone. Temporary G-CSF. HCT	Remission of lymphoma and ADA2 related symptoms
9	R169Q/ R169Q	43	45*	U	M	1. T-cell large granular lymphocytic (T-LGL) leukemia 2. Non-small cell lung carcinoma	Ciclosporin, cyclophosphamide. G-CSF *After DADA2 was diagnosed:* infliximab/methotrexate (only 1 dose), adalimumab/sirolimus. G-CSF Lung malignancy: palliative	Deceased
11	R169Q/ R169Q	29	33	U	H	T-cell large granular lymphocytic (T-LGL) leukemia	Prednisolone, azathioprine, methotrexate (only 1 dose), ciclosporin. G-CSF *After DADA2 was diagnosed*: infliximab/ciclosporin, adalimumab/ciclosporin, adalimumab/sirolimus, adalimumab/tacrolimus. G-CSF. Currently, HCT is considered	Infrequent treatable bacterial infections
12	R169Q/ P193L	46	46	0	H	Angio-immunoblastic T-cell lymphoma (AITL), EBV +	Chemotherapy (6 × CHOEP), subsequent autologous stem cell transplantation	Deceased. Initial complete remission followed by (presumed) late AITL relapse complicated by HLH. He most likely died of intracerebral hemorrhage due to thrombocytopenia
17	G47V/ c.973-2A > G	46	18	0	V	Basal cell carcinoma (BCC)	Surgical excision	Radical resection of BCC
20	R169Q/ R169Q	22	26	4	M	Basal cell carcinoma (BCC)	Surgical excision	Radical resection of BCC
23	R169Q/ R169Q	9	0.8**	0	M	1. AML related chloroma 2. Conjunctival granulomatous sarcoma 3. Schwannoma (benign)*** 4. Myxoid liposarcoma***	Chemotherapy (AML-15) and subsequent HCT Cyclophosphamide pulses and infliximab for vasculitis	Remission of AML and DADA2 related symptoms after HCT, but developed schwannoma and myxoid liposarcoma post-HCT (6 and 9 years post-HCT respectively)

U, unknown; R-CHP, rituximab, cyclophosphamide, doxorubicin, prednisone; CHOEP, cyclophosphamide, doxorubicin, etoposide, vincristine, prednisone. AML-15: cytarabine, daunorubicin, etoposide, anthracycline. * = Age at developing non-small cell lung carcinoma, T-LGL leukemia developed at an earlier stage. ** = Age at developing AML-related chloroma, other neoplasms developed later. *** = Developed post-HCT

Phenotype; V, inflammatory vasculopathy; H, hematologic manifestations; I, immunodeficiency; M, mixed. Mixed phenotype indicates a combination of two or three phenotypes (vasculopathy, hematologic manifestations, immunodeficiency)

### Secondary Hemophagocytic Lymphohistiocytosis (HLH)

Four patients developed HLH or an HLH-like episode during their disease course (patient 1, 10, 12 and 29). None of them was treated with TNFi prior to onset of HLH. Patient 1 presented at age 8 years (before the diagnosis of DADA2 was made) with EBV-infection complicated with fever, splenomegaly and sudden onset pancytopenia. Blood analysis showed elevated triglycerides, elevated soluble IL2 receptor, elevated ferritin and low fibrinogen. Eventually, fever and pancytopenia improved spontaneously and symptoms were interpreted as an acute EBV-infection complicated by an HLH-like episode.

Patient 10, known with familial neutropenia, hypogammaglobulinemia, recurrent infections and granulomatous inflammation, presented at age 53 years with jaundice and spiking fever up to 41 °C for 2–3 weeks. Blood analysis showed pancytopenia (Hb 6.2 mmol/L, thrombocytes 84 × 10^9^/L, neutrophils 0.4 × 10^9^/L) and elevated liver enzymes (ALAT up to 420 U/L and gamma GT up to 1281 U/L). Abdominal ultrasound showed progressive splenomegaly. Ferritin was markedly increased (up to 15.376 µg/L) and triglycerides were elevated (5.38 mmol/L). A bone marrow aspirate showed erythrophagocytosis. The patient was diagnosed with HLH, which proved refractory to corticosteroids and anakinra. Genetic evaluation showed biallelic pathogenic variants in the *ADA2* gene and infliximab was started, without sufficient effect on the HLH. The patient was subsequently treated with dexamethasone, etoposide, ciclosporin and later ruxolitinib, which induced clinical and biochemical improvement. In addition, rituximab was started for suspected auto-immune neutropenia. After the sixth cycle of treatment for HLH, the patient developed refractory septic shock and passed away.

Patient 12 presented at age 47 years with an angio-immunoblastic T-cell lymphoma (AITL), treated with chemotherapy and autologous HCT. Genetic evaluation for primary immunodeficiencies showed biallelic pathogenic ADA2 variants. During a period of (suspected) recurrence of lymphoma and EBV viremia (with negative serum EBV-IgM), he developed fever, splenomegaly, severe pancytopenia (Hb 3.8 mmol/L, thrombocytes 0 × 10^9^/L, leukocytes 0.1 × 10^9^/L), and elevated ferritin (87,000 μg/L), elevated triglycerides (4.09 mmol/L) and increased soluble IL2 receptor (> 7500 U/ml). The diagnosis EBV-induced HLH was made and the patient was treated with dexamethasone, etoposide, ciclosporin and rituximab. He was referred for allogeneic HCT, but passed away awaiting this therapy, presumably due to an intracerebral hemorrhage associated with severe thrombocytopenia.

Patient 29, known with recurrent infections (including pneumonia), granulomatous skin inflammation, aphthous stomatitis and diagnosis of seronegative optic neuritis at the age of 9 years, presented at age 10 years with 4 weeks of neutropenia and occasional febrile spikes. Genetic testing for an underlying immunological disease was deployed. In the 5^th^ week, she developed daily spiking fevers and progressive laboratory abnormalities matching with HLH, showing elevated ferritin (up to 13.542 μg/L), elevated triglycerides (up to 3.46 mmol/L), mildly decreased fibrinogen (1.2 g/L) and pancytopenia (Hb 6,4 mmol/L, thrombocytes 37 × 10^9^/L and neutrophils 0.0 × 10^9^/L). PET-CT showed increased activity in bone marrow, spleen and lymph nodes. Methylprednisolone-pulse therapy was started with adding of TNF-inhibition, after genetic confirmation of DADA2 at day 3 of the pulses. Nonetheless, she developed respiratory insufficiency due to pulmonary infiltrates and opacities, finally evolving into extensive pulmonary edema. Despite adding an IL1-inhibitor (high-dose anakinra), the patient developed multi-organ failure in the next 3 days and died due to refractory ventricular fibrillation.

### Rare Manifestations

Rare manifestations of DADA2 in this cohort included pure red cell aplasia (patient 2 and 15), T-LGL leukemia (patient 9 and 11), IBD-like disease (patient 4 and 7), congenital aplastic anemia (patient 6), optic neuritis (patient 6 and 29), progressive liver fibrosis presumably due to non-infectious nodular regenerative hyperplasia (patient 1), venous thrombosis (patient 10 and 18), congenital chylothorax (patient 20), transient acute liver failure (patient 8), growth hormone deficiency (patient 1 and 2, siblings) and specific polysaccharide antibody deficiency syndrome (patient 16).

### ADA2 Enzyme Activity and Phenotype

**Fig. 1:**
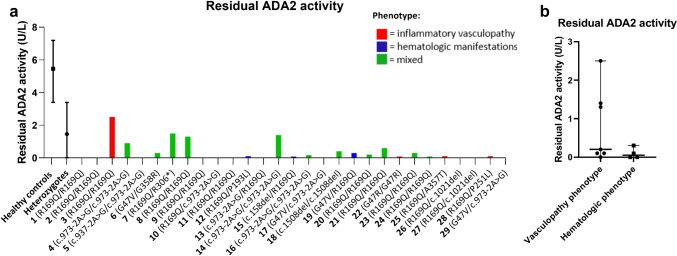
**A** Residual ADA2 activity in a cohort of 29 DADA2 patients before initiation of TNFi. Empty columns indicate a missing value. Mixed phenotype indicates a combination of two or three phenotypes (vasculopathy, hematologic manifestations, immunodeficiency). Residual ADA2 activity in healthy controls was based on the mean residual activity and range of 11 healthy controls (mean 5.46 U/L, range 3.4–7.2 U/L). Residual ADA2 activity in heterozygotes was based on the mean residual activity and range of 15 carriers of pathogenic ADA2 variants (mean 1.45 U/L, range 0.0–3.4 U/L). **B** Residual ADA2 activity in DADA2 patients with a vasculopathy phenotype (with or without immunodeficiency) versus hematologic phenotype (with or without immunodeficiency). * Only residual ADA2 activity values measured before initiation of TNFi are used

Residual ADA2 enzyme activity before initiation of TNFi was measured in 18 patients (Fig. 1A and Supplementary Table 3A). Residual ADA2 enzyme activity after initiation of TNFi was measured in 9 patients (Supplementary Table 3B), but was not used in analyses. When comparing residual ADA2 activity between patients with stroke (*n* = 12; 2 missing values) and patients without stroke (*n* = 16; 9 missing values), we found no significant differences. Similarly, when comparing patients with a hematologic phenotype (with or without concomitant immunodeficiency) (*n* = 9; 5 missing values) to patients with a vasculopathy phenotype (with or without immunodeficiency) (*n* = 11; 4 missing values), we found no significant difference in residual ADA2 enzyme activity (Fig. 1B). However, it is worth mentioning that all four patients in this cohort with residual ADA2 levels above 1 U/L (patient 3, 7, 8 and 14) exhibited inflammatory vasculopathy features. Remarkably, patient 22 (with a homozygous pathogenic G47R variant) and patient 25 (with compound heterozygous pathogenic R169Q/A357T variants), both expressed extremely low ADA2 enzyme activity (0–0.1 U/L, respectively), while the pathogenic variants G47R and A357T were previously described to express relatively high residual ADA2 activity levels [6, 22].

### Genotype and Phenotype

All patients had biallelic pathogenic variants in the *ADA2* gene (Table 2)*.* The most common pathogenic variant in this cohort was homozygous c.506G > A p.(Arg169Gln) (34.5%), followed by homozygous c.973-2A > G p.(?) (13.8%) (Tables 1 and 2). The c.1508del p.(Lys503fs) pathogenic variant of patient 18 has not been reported before. All patients with a homozygous c.973-2A > G p(?) pathogenic variant exhibited vasculopathy features (sometimes in combination with immunodeficiency or hematologic manifestations), whereas patients with homozygosity for the c.506G > A p.(Arg169Gln) pathogenic variant exhibited highly variable phenotypes including severe hematologic manifestations without vasculopathy (such as bone marrow failure and PRCA). We found no significant differences in residual ADA2 activity between patients with these two pathogenic variants (data not shown).

### Treatment

**Fig. 2:**
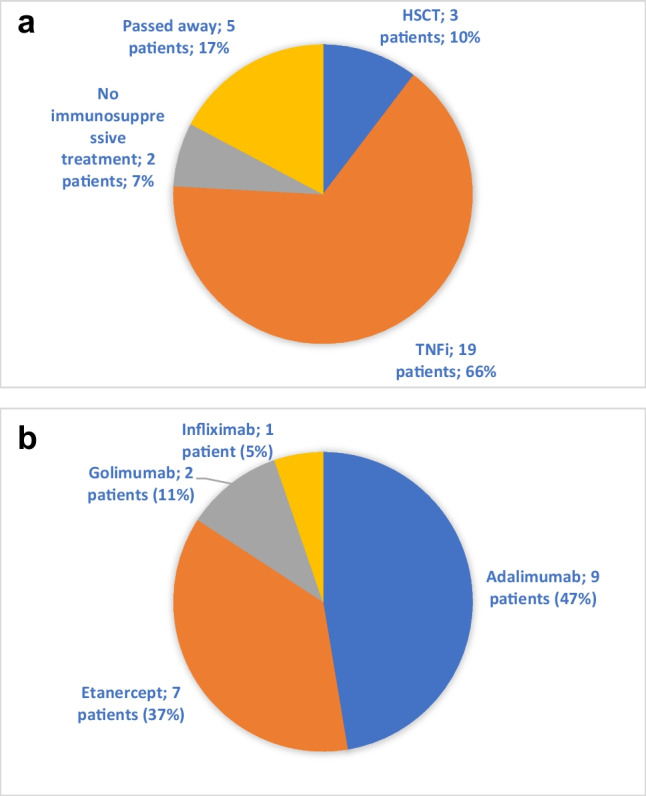
**A** Current treatment in a cohort of 29 DADA2 patients. **B** Currently prescribed TNF-inhibitors in 19 DADA2 patients

Figure 2A shows the current treatment of all patients (*n* = 29). A total of 21 patients are currently alive and have not undergone HCT. Of them, 19 are treated with TNFi, including etanercept, infliximab, golimumab and adalimumab (Figs. 2A and B). Three patients received concomitant methotrexate (MTX) to prevent TNFi antibodies, of which one developed neutralizing antibodies against adalimumab. One patient started MTX after neutralizing adalimumab antibodies were found, followed by normalization of serum adalimumab levels. Four patients in this cohort received IL-1 inhibition, of whom two during active HLH. In the total cohort (*n* = 29), four patients never received TNFi [2, 12, 13, 19]. Patient 2 underwent HCT before DADA2 was recognized and patients 13 and 19 declined TNFi for personal reasons. Patient 12 was awaiting HCT and TNF-inhibition was under consideration when the patient passed away.

Table 5 and Supplementary Table 4 show DADA2-related symptoms before and after initiation of TNFi. All patients showed amelioration (and often reversal) of cutaneous symptoms (except eczema) with TNFi, although cutaneous vasculitis and other skin symptoms occasionally showed mild flare-ups in some patients. Even though none of the patients developed an MRI-confirmed stroke after adequate disease control with TNFi, two patients had episodes suspected of recurrent ischemia. During a period of poor response to infliximab, patient 3 developed a flare-up of skin ulcers and three inflammatory lesions in cerebro causing vertigo, gait disorder and neglect. Repeated MRI’s were compatible with a vascular origin of the lesions and a brain biopsy showed lymphocytic infiltration, consistent with possible infarction. In addition, patient 18 developed gait disorder, diplopia and dysarthria while on TNF-inhibition without abnormalities on MRI. Other symptoms that developed under TNF-inhibition included progressive liver cirrhosis, most probably due to nodular regenerative hyperplasia, and peripheral facial palsy without abnormalities on MRI (both patient 1).

**Table 5:** Clinical manifestations in DADA2 patients before and after start TNF-inhibition. Column ‘Before’ shows clinical manifestation before the onset of TNF-inhibition and column ‘After’ shows clinical manifestations that developed or did not improve under TNF-inhibition

Clinical manifestations	Before	After
Cutaneous involvement (except eczema)	16	0
Eczema	3	4
Fever	8	0
Stroke	10	2*
PAN-like rash or other cutaneous vasculitis	10	0
Arthralgia/artritis	6	2
IBD-like symptoms	2	0
Aphthous stomatitis	9	2
Anemia	5	5
Thrombocytopenia	4	4
Neutropenia	7	5
Lymphopenia	7	6
Hypogammaglobulinemia	10	10

* = Both cases are suspected of stroke. One patient developed lesions suspected of stroke during a period of poor response to TNFi (infliximab), one patient developed clinical symptoms suspected of stroke without MRI-abnormalities

TNFi had no effect on cytopenia in most patients. Cytopenia in patient 1 only reversed after splenectomy. Patient 7 showed improvement of anemia and lymphocyte-driven immune-mediated neutropenia after starting infliximab, prednisone and ciclosporin. It is impossible to determine the role of TNF-inhibition in the reversal of cytopenia in this patient, as all medication was started concomitantly and induced remission of disease. Patients 16 and 21 developed anemia and/or thrombocytopenia while using TNFi, suggesting that TNFi do not protect against developing cytopenia or that cytopenia is a side effect of TNFi.

### Allogeneic Hematopoietic Cell Transplantation

Three patients in this cohort (patient 2, 7 and 23) underwent HCT for various indications. Patient 2 underwent HCT because of refractory pure red cell aplasia (PRCA) and transfusion-associated iron overload. Patient 7 was transplanted after complete remission of diffuse large B-cell lymphoma was achieved with chemotherapy and DADA2 symptoms were well controlled with infliximab, ciclosporin and prednisone. Transplantation indications were the increased risk of recurrent lymphoma and the complexity of his DADA2 phenotype. Patient 23 underwent HCT because of pancytopenia, including absence of B cells, after AML treatment. As described, patient 23 developed a schwannoma of the ischiadic nerve and a myxoid liposarcoma in the upper leg after HCT. The other two patients are doing well after HCT and their pre-existent DADA2 phenotype resolved. Two out of three patients were treated with TNFi before transplantation (patient 7 and 23). Patient 2 did not receive TNFi prior to HCT, because DADA2 was not yet recognized. Currently, patient 15 is scheduled for HCT with severe anemia and refractory neutropenia as indications.

### Mortality

Five patients passed away in this cohort (17.2%). As described, patient 9 died of a pulmonary malignancy, patient 10 died of septic shock during treatment for HLH and patient 12 most likely died of an intracerebral hemorrhage due to severe thrombocytopenia as a complication of HLH. Patient 21 developed bone marrow failure refractory to TNFi, followed by recurrent infections, organizing pneumonia and fatal neutropenic enterocolitis. Patient 29 passed away due to HLH and the resulting refractory hyperinflammatory state leading to multi-organ failure. The 3 of the 5 patients who died from HLH or HLH-associated complications were recently diagnosed with DADA2 and TNFi were not started before the onset of HLH. Patient 9 tried TNFi for a short time with no effect on cytopenia and discontinued due to infectious complications. Patient 21 developed bone marrow failure while using TNFi and was still on TNFi when he got admitted with neutropenic enterocolitis, which became fatal.

## Discussion

This study describes the clinical, genetic and laboratory findings of 29 Dutch DADA2 patients with various pathogenic variants in the *ADA2* gene. We describe the occurrence of HLH and HLH-like episodes as a life-threatening disease complication and report a relatively high incidence of malignancies and a relatively high mortality rate.

Five patients in this cohort developed a hematologic malignancy: EBV-positive lymphoma, two cases of T-LGL leukemia, AML-related chloroma and angio-immunoblastic T-cell lymphoma (AITL). T-LGL and EBV-related lymphoproliferation have been described before in DADA2 patients [24, 25]. AITL has never been described in DADA2 patients but is considered EBV-related, even though the role of EBV in pathophysiology is not yet elucidated [26]. The presumed pathogenesis of T-LGL leukemia is clonal LGL expansion due to chronic antigen stimulation [27]. As DADA2 is an auto-inflammatory disease, T-LGL might be associated with prolonged exposition to chronic inflammation. The reported EBV-related lymphomas may indicate an indirect role for ADA2 in defense against viral infections. Arts et al. described a case series of two DADA2 patients with recalcitrant mollusca as their initial or predominant presentation. They hypothesized that DADA2 patients have an increased susceptibility to double stranded DNA viruses, such as human papillomavirus (HPV) and herpes viridae [28]. Indeed, EBV-related malignancies, herpes labialis, varicella zoster infections and warts were reported in this cohort (data not shown). Other factors that may contribute to the development of malignancies in DADA2 are persistent inflammation, genotype and immunosuppressive treatment. However, the occurrence of malignancy constituted the initial presentation in three patients (patient 7, 12 and 23) who were not on immunosuppressive treatment yet. At this moment, it is unclear whether the malignancies we here report are causally related to DADA2, but causality seems more plausible for hematological malignancies than for solid tumors. It is important to note that the presence of severe viral infections and (EBV-related) malignancies should alert to the possibility of DADA2. Moreover, clinicians should be aware that DADA2 patients are at risk of developing malignancies and further research should determine whether routine screening for malignancies is indicated.

In this cohort, three patients presented with fulminant secondary HLH and one patient with an HLH-like episode. Secondary HLH, has occasionally been described in DADA2 patients [1, 14, 29]. The incidence of HLH in our cohort was high and 3 of 4 patients with HLH passed away. Of note, none of these patients was treated with TNFi before the onset of HLH, and it is unknown whether TNFi could have prevented the onset. Successful treatment of HLH with a TNF-inhibitor (etanercept) has been reported; however, other studies describe that TNFi might induce or worsen HLH [30–33]. Usually, TNFi have no role in HLH-treatment; instead, depending on the suspected HLH-trigger, treatment regimens contain corticosteroids or biologicals including IL1- or IL6-inhibitors [34]. It is suggested that during HLH, hemophagocytic macrophages express a classical pro-inflammatory M1 phenotype [35] and this may explain a link between DADA2 and HLH, as absence of ADA2 results in skewing of macrophages to the pro-inflammatory M1 phenotype [1, 10]. In addition, Lee et al. found elevated ADA2 levels in systemic juvenile arthritis patients with MAS, a form of secondary HLH [36]. Combined with the high prevalence of HLH in our DADA2 cohort, one could speculate that DADA2 may lower the threshold for HLH and may be associated with a more severe course once HLH is present. It is important for clinicians to consider DADA2 in patients presenting with HLH and to be aware of HLH as a possible (fatal) complication of DADA2.

Based on previous reports on TNFi in DADA2 patients presenting with (cerebral) vasculopathy, TNFi are the first choice treatment in DADA2. In our cohort, TNFi were effective in preventing and treating vasculopathy-associated features such as fever and PAN-like skin disease. None of our patients developed MRI-confirmed stroke after adequate disease control with TNFi, but recurrent ischemia cannot be completely ruled out in two patients. In most patients, cytopenia did not improve after initiation of TNFi. This is in line with most previous studies [6, 17], but cytopenia and even bone marrow failure responsive to TNFi have occasionally been described [37, 38], and pancytopenia and PRCA responsive to ciclosporin has also been reported [38, 39]. Our cohort describes variable responses to ciclosporin, including one patient whose cytopenia improved after treatment with infliximab, ciclosporin and prednisone, but also two T-LGL leukemia patients without improvement of neutropenia on ciclosporin. We also noted that some patients were treated with several types of TNFi during their disease course due to reduced effectiveness or side effects, underscoring the need for alternative therapeutic options as there is only a limited number of TNFi [14]. HCT is considered an effective and definitive treatment for all DADA2 phenotypes [3, 40]. HCT is mainly performed for severe phenotypes unresponsive to TNFi, such as severe hematological manifestations and immunodeficiency, and may be considered in patients who have developed and received treatment for malignancy. In this cohort, HCT was performed in three patients for various indications. Two patients are doing well after successful HCT without DADA2-related complaints. One patient developed a schwannoma and myxoid liposarcoma after HCT. It is unknown whether these malignancies are related to DADA2 as this patient showed normal serum ADA2 activity after HCT.

The observation that TNFi have limited effect in hematologic manifestations and immunodeficiency raises important clinical questions, such as whether TNFi should be initiated in patients expressing only a hematologic phenotype, especially given the increased susceptibility to infections that can be seen in these patients. This cohort confirms that stroke can occur later in the disease course, even in patients presenting with a ‘purely’ hematologic phenotype. For example, we describe a patient who presented with congenital aplastic anemia and developed cranial nerve deficits and humoral immunodeficiency 20 years later. At age 30, she suffered a stroke, showing that phenotypes can expand within patients, even after many years of apparently stable disease. Another clinical dilemma is whether TNFi should be prescribed to all asymptomatic patients for stroke prevention. This cohort confirms that stroke can be the first (recognized) symptom of DADA2, suggesting that TNFi should be offered to all symptomatic DADA2 patients regardless the severity of complaints. Whether it should be offered to adults with DADA2 who have never been symptomatic remains unsolved, as this cohort is too small to address this question. More (prospective) studies and a better understanding of the pathophysiology of DADA2 are urgently needed to optimize recommendations for DADA2 patients with variable disease manifestations.

In line with Lee et al., all patients with homozygous pathogenic c.973-2A > G p.(?) variants in this cohort showed vasculopathy features, while patients with pathogenic c.506G > A p.(Arg169Gln) variants showed highly variable phenotypes [6]. Patients with identical genotypes exhibited different phenotypes and variable residual ADA2 activity, as previously reported by Van Montfrans et al. This suggests that factors other than the specific ADA2 variant are important for residual enzyme activity and clinical course [8]. In this cohort, we found no difference in residual ADA2 enzyme activity in hematologic patients compared to vasculopathy patients, contrary to what has been previously described by Lee et al. On the other hand, using an in vitro overexpression system with 20-fold higher ADA2-activity than in vivo, Lee et al. predicted that the differences in residual activity they found in vitro will not be observable in vivo [6]. Plasma ADA2-activity measurement in vivo is probably not sufficiently sensitive to detect small differences between phenotypes, and is therefore unlikely to be useful in predicting the clinical course in individual patients. However, it should be noted that the predominant genetic variant in our cohort (c.506G > A p.(Arg169Gln)) has been reported to express low ADA2 residual activity levels compared to other pathogenic variants. This might limit the conclusions that can be drawn from this analysis. Lastly, it is noteworthy that ADA2 enzyme activity decreased in most patients after initiation of TNFi. It can be speculated that TNFi inhibit macrophages and thereby reduce the macrophage-derived production of the ADA2 enzyme [23].

A strength of our study is that it provides an in-depth overview of almost all known DADA2 patients in the Netherlands. At the same time, it is a limitation that some Dutch patients still may be undiagnosed due to, for example, incomplete disease penetrance, variable age of disease onset and pleiotropic manifestations [22]. A recent study estimated a DADA2 prevalence of 1 in 222,164 individuals worldwide, possibly even higher in Northern Europe (22), which would imply that we have so far diagnosed approximately half of all Dutch DADA2 patients. Other limitations include the retrospective nature of this study and the relatively small size of the cohort, limiting the power of the statistical analyses.

In conclusion, we report a high incidence of HLH in DADA2 patients with a high mortality rate (75%). We found a relatively high incidence of hematologic malignancies, of which the relationship to DADA2 needs further investigation. This cohort confirms that TNFi are effective for stroke prevention and treatment of vasculopathy-associated symptoms, and underscores the clinical need for improved treatment of hematologic manifestations.

## Supplementary Information

Below is the link to the electronic supplementary material.

Supplementary file1 (DOCX 69 KB)

## Data Availability

Aggregated datasets generated and/or analyzed during the current study are available from the corresponding author on reasonable request.
