# Supplementary material for: Clinical Symptoms, Laboratory Parameters and Long-Term Follow-up in a National DADA2 Cohort
Source: J Clin Immunol. 2023 Jun 5;43(7):1581–96. doi: 10.1007/s10875-023-01521-8 (PMC10499949; doi:10.1007/s10875-023-01521-8)
Supplement: Supplementary file 1 — Supplementary file1 (DOCX 69 KB) [file 10875_2023_1521_MOESM1_ESM.docx]

**Supplementary Table 1: Clinical manifestations in a cohort of DADA2 patients (N=29)**

|  | **1*** | **2** | **3** | **4** | **5** | **6** | **7** | **8** | **9** | **10*** | **11** | **12*** | **13** | **14** | **15** | **16** | **17** | **18** | **19** | **20** | **21** | **22** | **23** | **24** | **25** | **26** | **27** | **28** | **29*** |
| --- | --- | --- | --- | --- | --- | --- | --- | --- | --- | --- | --- | --- | --- | --- | --- | --- | --- | --- | --- | --- | --- | --- | --- | --- | --- | --- | --- | --- | --- |
| Age at inclusion | 20 | 23 | 56 | 15 | 11 | 56 | 29 | 19 | 46 (†) | 52 (†) | 33 | 47 (†) | 51 | 21 | 49 | 25 | 47 | 59 | 26 | 29 | 27 (†) | 33 | 15 | 13 | 16 | 18 | 10 | 6 | 10  (†) |
| Age at presentation | 1 | 0 | 6 | 1 | 1 | 19 | 6 | 8 | 28 | 18 | 11 | 3 | 2 | 6 | 1 | 14 | 20 | 19 | 18 | 3 | 1 | 4 | 0.8 | 5 | 16 | 7 | 4 | 5 | 0 |
| Gender (Male= M, Female= F) | M | M | M | M | M | F | M | F | F | M | F | M | M | M | F | F | F | M | F | M | M | M | F | F | M | M | F | M | F |
| Recurrent fever | X |  |  | X | X |  |  | X |  | U |  | X | X | X |  | U | X | U |  |  |  |  | X | U | X | X |  |  | X |
| Splenomegaly | X | X | X | X |  |  | X |  | X | X |  | U | X | U | U | U | X | X | X | X | X | X | X |  | X | X |  |  | U |
| Hepatomegaly | X | X | X | X |  |  | X |  | X | U |  | U |  | U | U | U | U | U | X | X |  | X | X |  | U |  |  | X |  |
| Stroke (ischemic or hemorraghic) | X |  | X |  |  | X | X | X |  |  |  | X** |  |  |  |  |  | X |  | X | X | X | X |  |  |  | X | X |  |
| Skin |  |  |  |  |  |  |  |  |  |  |  |  |  |  |  |  |  |  |  |  |  |  |  |  |  |  |  |  |  |
| Livedo reticularis | X | X | X |  |  |  |  |  |  | X |  |  |  | X |  | X | X |  |  |  |  | X |  |  | X |  |  | X |  |
| Livedo racemosa |  |  |  |  |  |  |  |  |  |  |  |  |  |  |  | X |  |  |  | X |  |  |  |  |  | X |  |  |  |
| Livedoid vasculopathy |  |  | X |  |  |  |  |  |  |  |  |  |  |  |  |  |  |  |  |  |  |  |  |  |  |  |  |  |  |
| Skin ulcers |  |  | X |  |  |  |  |  |  |  |  |  |  |  |  |  |  |  |  |  |  |  |  |  |  |  |  |  |  |
| Erythema nodosum | X |  |  | X |  |  |  |  |  |  |  |  | X | X |  |  |  |  |  | X |  | X |  |  |  |  | X |  |  |
| Rash |  | X |  | X | X |  | X |  |  |  |  | X |  |  |  |  |  |  |  |  | X |  |  |  |  |  |  |  |  |
| Eczema | X | X |  |  |  |  |  |  |  |  |  |  |  |  |  |  |  |  |  |  | X | X | X | X |  |  | X | X |  |
| Skin abnormalities | X |  |  |  |  |  |  |  |  |  |  |  |  |  |  |  | X | X |  | X | X |  | X |  |  |  |  |  | X^3^ |
| Cutaneous vasculitis |  |  |  |  |  |  |  |  |  |  |  |  |  |  |  |  |  |  |  |  |  |  |  |  |  |  |  |  |  |
| Polyarteriitis nodosa |  |  |  |  |  |  |  |  |  |  |  |  |  | X |  | X |  | X |  | X |  |  |  |  |  |  |  | X |  |
| Other documented vasculitis |  |  | X |  |  | X^1^ | X^2^ |  |  |  |  |  |  |  |  |  |  |  |  | X | X | X^1^ |  |  |  |  |  |  |  |
| Systemic vasculitis |  |  |  |  |  |  |  |  |  |  |  |  |  |  |  |  |  |  |  |  |  |  | X |  |  |  |  |  |  |
| Gastro-intestinal |  |  |  |  |  |  |  |  |  |  |  |  |  |  |  |  |  |  |  |  |  |  |  |  |  |  |  |  |  |
| Feeding difficulties |  |  |  |  |  |  |  |  |  |  |  |  |  |  |  |  |  |  |  | X |  |  | X |  |  |  |  |  |  |
| IBD-like symptoms |  |  |  | X |  |  | X |  |  |  |  |  |  |  |  |  |  |  |  |  |  |  |  |  |  |  |  |  |  |
| Aspecific abdominal pain | X | X |  |  | X |  |  | X |  |  |  |  |  |  |  |  |  |  |  |  |  |  | X |  |  |  |  |  |  |
| Acute liver failure |  |  |  |  |  |  |  | X |  |  |  |  |  |  |  |  |  |  |  |  |  |  |  |  |  |  |  |  |  |
| Nodular regenerative hyperplasia | X |  |  |  |  |  |  |  |  |  |  |  |  |  |  |  |  |  |  |  |  |  |  |  |  |  |  |  |  |
| Aphthous stomatitis | X |  |  | X | X | X |  | X | X |  | X |  |  |  |  |  |  |  |  |  |  |  | X |  | X |  | X |  | X |
| Cardiovascular |  |  |  |  |  |  |  |  |  |  |  |  |  |  |  |  |  |  |  |  |  |  |  |  |  |  |  |  |  |
| Hypertension |  |  |  |  |  | X |  |  |  |  |  |  |  |  |  |  |  |  |  |  |  | X | X |  |  |  |  |  |  |
| Cardiomyopathy (dilative) |  |  |  |  |  |  |  |  |  |  |  |  |  |  |  |  |  |  |  |  |  |  | X |  |  |  |  |  |  |
| Recurrent infections |  |  |  |  |  |  |  |  |  |  |  |  |  |  |  |  |  |  |  |  |  |  |  |  |  |  |  |  |  |
| Upper respiratory tract infections | X |  |  |  |  | X |  | X | X | X | X | X | X |  | X |  |  | X |  |  | X |  | X | X |  |  |  |  | X |
| Lower respiratory tract infections |  |  |  |  |  |  |  |  | X | X |  |  |  |  |  |  |  | X |  |  |  |  | X | X |  | X |  |  | X |
| Skin infections |  |  | X |  |  | X |  |  |  |  | X |  |  |  |  |  | X |  |  |  | X |  | X | X |  |  |  |  |  |
| Gastro-intestinal infections |  |  |  |  |  |  |  |  |  |  |  |  |  |  |  |  |  |  |  |  |  |  |  |  |  |  |  |  |  |
| Urinary tract infections |  |  |  |  |  |  |  |  |  |  |  |  |  |  |  |  |  |  |  |  |  |  |  |  |  |  |  |  |  |
| Joints and extremities |  |  |  |  |  |  |  |  |  |  |  |  |  |  |  |  |  |  |  |  |  |  |  |  |  |  |  |  |  |
| Arthralgia | X |  |  | X | X |  |  | X |  |  | X | X |  |  |  |  |  |  |  |  |  |  | X |  | X | X |  |  |  |
| Arthritis |  |  |  |  |  |  |  |  |  |  |  |  |  |  |  |  |  |  |  |  |  |  | X |  |  |  |  |  |  |
| Myalgia | X |  |  |  |  |  |  |  |  |  | X | X |  |  |  |  | X |  |  |  |  |  |  |  | X |  |  |  |  |
| Unexplained pain lower extremities | X |  | X |  |  |  |  |  |  |  |  |  |  |  |  |  |  |  |  | X |  |  |  |  |  | X | X |  |  |
| Raynaud phenomenon | X |  |  |  |  | X |  |  |  |  |  |  |  |  |  |  |  |  |  | X |  |  |  |  |  |  |  |  |  |
| Malignancy (all types, including BCC) |  |  | X |  |  |  | X |  | X |  | X | X |  |  |  |  | X |  |  | X |  |  | X |  |  |  |  |  |  |
| Hematology and coagulation |  |  |  |  |  |  |  |  |  |  |  |  |  |  |  |  |  |  |  |  |  |  |  |  |  |  |  |  |  |
| Anemia | X | X |  |  |  |  | X |  |  | X | X | X |  |  | X | X |  |  | X |  | X | X | X | X |  |  |  |  | X |
| Thrombocytopenia | X |  |  |  |  |  | X |  | X | X |  | X |  |  |  |  |  | X | X |  | X |  | X | X |  |  |  |  | X |
| Leukopenia | X |  |  | X |  | X | X | X | X | X | X | X | X |  | X |  |  | X | X |  | X |  | X |  |  |  |  |  | X |
| Neutropenia | X | X |  | X |  |  | X |  | X | X | X | X | X |  | X |  |  |  | X |  | X |  | X |  |  |  |  |  | X |
| Lymphopenia | X |  |  |  |  |  | X |  | X | X |  | X |  |  | X |  |  | X |  | X | X |  |  |  |  |  |  |  | U |
| Hypogammaglobulinemia | X | X |  |  |  | X | X | X | X | X |  | U |  | X |  |  |  | X |  | X | X |  | X | X |  | X |  |  |  |
| Hemophagocytic lymphohistiocytosis (HLH) | X |  |  |  |  |  |  |  |  | X |  | X |  |  |  |  |  |  |  |  |  |  |  |  |  |  |  |  | X |
| Phenotype | M | M | V | M | V | M | M | M | M | M | H | H | M | M | H | M | V | M | H | M | M | V | M | M | V | M | V | V | M |

**Hematology and coagulation during HLH; U= unknown; IBD= inflammatory bowel disease; BCC= basal cell carcinoma; Phenotype: V= inflammatory vasculopathy, H=hematologic manifestations, M= mixed. Mixed phenotype indicates a combination of two or three phenotypes (vasculopathy, hematologic manifestations, immunodeficiency).*

*X^1^ = lymphocytic vasculitis; X^2^ = leucocytoclastic vasculitis;* *X^3^ = skin biopsy showed multifocal interstitial granulomatous inflammation; X**: presumed intracerebral hemorrhage due to thrombocytopenia, but CT-scan was not performed.*

**Supplementary Table 2A: Laboratory values (#) in a cohort of DADA2 patients (N=29)**

| **Patient** | **Hemoglobin (normal range per age group), mmol/l** | **Thrombocytes, 150-450 x 10^9^/l** | **Leukocytes (normal range per age group), 10^9^/l** | **Neutrophils (normal range per age group), 10^9^/l** | **Lymphocytes (normal range per age group), 10^9^/l** | **Immunoglobulins; IgM, 0.28-2.4 g/l** | **Immunoglobulins; IgG, 5.2-15.6 g/l** | **Immunoglobulins; IgA, 0.7-3.6 g/l** |
| --- | --- | --- | --- | --- | --- | --- | --- | --- |
| **1** | 6.3 (8.6-10.7) | 39 | 1.1 (4.5-13.0) | 0.39 (1.8-8.0) | 0.48 (1.2-5.2) | 0.19 | 2.36 | 0.11 |
| **2** | 4.9 (7.4-8.7) | 365 | 7.8 (5.5-15.5) | 0.78 (1.5-8.5) | 5.61 (2.0-8.0) | 0.3 | 3.6 | 0.6 |
| **3** | 8.3 (8.6-10.7) | 402 | 9.8 (4.0-10.0) | 6.78 (1.6-8.3) | 1.78 (0.8-4.0) | 0.71 | 9.89 | 2.7 |
| **4** | 8.2 (7.4-9.0) | 237 | 3.2 (4.5-13.0)^##^ | 1.48 (1.8-8.0)^##^ | 1.05 (1.2-5.2) | 0.72 | 8.67 | 0.82 |
| **5** | 7.2 (7.4-9.0) | 328 | 6.1 (4.5-13.5) | 3.49 (1.8-8.0) | 1.65 (1.5-6.5) | 0.34 (0.13-1.6) | 7.66 | 0.77 (0.62-3.0) |
| **6** | 8.6 (7.4-9.6) | 211 | 2.5 (4.0-10.0) | U | U | 0.41 (0.4-2.3) | 4.69 (7.0-16.0) | 0.61 (0.7-4.0) |
| **7** | 6.2 (8.6-10.7) | 101 | 0.2 (4.0-10.0) | 0 (1.6-8.3) | U | 0.09 (0.4-2.3) | 5.63 (7.0-16.0) | 0.18 (0.7-4.0) |
| **8** | 7.7 (7.4-9.6) | 213 | 4.9 (4.5-13.0) | 2.69 (1.8-8.0) | 1.51 (1.2-5.2) | <0.1 | 3.6 | 0.2 |
| **9** | 7.4 (7.5-10.0) | 104 | 8.4 (4.0-10.0) | 8.3 (1.8-7.0)** | 0.51 (1.0-3.5) | 0.1 (0.4-2.3)* | 15* | <0.1 (0.6-4.0)* |
| **10***** | 6.5 (8.5-11.0) | 92 | 0.6 (4.0-10.0) | U | 0.01 (1.0-3.5) | U | 4.5 (7.0-16.0)* | U |
| **11** | 6.2 (7.5-10.0) | 172 | 2.6 (4.0-10.0) | 0.95 (1.8-7.0) | 1.28 (0.8-3.2) | 1.6 (0.4-2.3) | 10 (7.0-16.0) | 1.9 (0.7-4.0) |
| **12****** | 7.3 (8.5-11.0) | 265 | 9.2 (4.0-10.0) | 8.63 (1.8-7.0) | 0.38 (0.8-3.2) | U | U | U |
| **13** | 10.3 (8.5-11.0) | 147 | 3.0 (4.0-10.0) | 0.42 (1.8-7.0)** | 1.77 (0.8-3.2) | 0.4 (0.4-2.3) | 7.1 (7.0-16.0) | 1.7 (0.7-4.0) |
| **14** | 7.9 (8.0-10.4) | 294 | 5.4 (4.0-10.0) | 2.85 (1.5-8.0) | 1.8 (1.0-3.5) | 0.14 (0.54-1.93) | 5.59 (7.3-16) | 0.57 |
| **15** | 5.9 (7.4-9.9) | 204 | 1.2 (4.0-10.0) | 0.25 (1.5-8.0) | 0.84 (1.0-3.5) | U | 8.62 | U |
| **16** | 6.7 (7.4-9.9) | 201 | 5.9 (4.0-10.0) | 3.18 (1.5-8.0) | 2.2 (1.0-3.5) | 0.49 | 10.62 | 1.92 |
| **17** | 8.9 (7.4-9.9) | 286 | 4.4 (4.0-10.0) | U | U | 0.21 | 10.6 | 1.66 |
| **18** | 9.6 (8.5-11.0) | 100 | 3.9 (4.0-10.0) | 3.26 (1.5-8.0) | 0.34 (1.0-3.5) | 0.16 | 3.9 | 0.63 |
| **19** | 6.6 (7.4-9.9) | 91 | 1.4 (4.0-10.0) | 0.22 (1.5-8.0) | U | 0.6 | 16.34 | U |
| **20** | 8.1 (8.6-10.7) | 179 | 4.3 (4.0-10.0) | 2.78 (1.6-8.3) | 0.57 (0.8-4.0) | <0.03 | 4.2 | 0.08 |
| **21^*/^** | 7.7 (7.4-8.7) | 270 | 0.9 (4.0-10.0) | 0.33 (1.6-8.3) | 0.43 (0.8-4.0) | <0.03 | 2.2 | 0.09 |
| **22** | 5.3 (8.5-10.5) | 300 | 6.6 (4.0-10.5) | 4.28 (1.8-7.2) | 1.45 (1.5-4.0) | 0.97 (0.4-2.3) | 14.7 (7-16) | 5.04 (0.7-4) |
| **23** | 4.3 (7.4-8.7) | 120 | 2.7 (5.5-15.5) | 0.9 (1.5-8.5) | 22.9 (2.0-8.0) | 0.09 | 3.54 | 0.34 |
| **24^**/^** | 8.1 (7.0-9.2) | 261 | 6.0 (4.2-9.4) | 3.51 (1.82-7.47) | 1.76 (1.16-3.33) | 0.2 | 4.6 | 0.3 |
| **25** | 7.5 (7.4-9.0) | 364 | 6.7 (4.0-10.0) | 3.82 (1.5-8.0) | 1.77 (1.0-3.5) | 0.3 | 13.5 | 2.4 |
| **26** | 8.0 (8.5-11.0) | 175 | 4.7 (4.0-10.0) | 2.14 (1.5-8.0) | 1.75 (1.0-3.5) | 0.10 | 7.0 | 0.57 |
| **27** | 7.6 (7.0-9.2) | 230 | 4.0 (4.0-10.5) | 1.40 (1.5-8.0) | 1.89 (1.5-4.0) | 0.99 | 8.6 | 1.05 |
| **28** | 7.3 (6.5-10.0) | 235 | 5.6 (5.0-15.0) | 3.3 (1.5-8.0) | 1.5 (1.5-4.0) | 0.18 | 11.1 | 0.96 |
| **29^***/^** | 7.4 (7.0-9.2) | 288 | 2.3 (4.0-10.5) | 0.51 (1.5-8.0) | 1.47 (1.5-4.0) | 0.56 | 6.17 | 0.27 |

*#= Laboratory values were obtained at representative time-points in the disease course*; ##= (mild) leukopenia and neutropenia were progressive **= immunoglobulins while using IVIG; **= neutrophils while using G-CSF. ***= Laboratory values were collected during HLH. ****= Laboratory values were collected after chemotherapy, but before HLH developed. */= Laboratory values were collected before the patient developed bone marrow failure. **/= Laboratory values were collected after the episodes of AIHA (auto-immune hemolytic anemia) and ITP (immune thrombocytopenic purpura). ***/= Laboratory values were collected before HLH developed. U= unknown*

*Note: Patients with low CD19+ B-cells without hypogammaglobulinemia are not considered immunodeficiency phenotype. Patients with solely decreased IgM or IgA are not counted as hypogammaglobulinemia.*

**Supplementary Table 2B: Additional laboratory values (#) in a cohort of DADA2 patients (N=29)**

| **Patient** | CD4+ T cells^a^ (normal range for age), cell count/mm3 | Naive CD4+T cells^a^ (normal range for age), % | CD8+ T cells^a^ (normal range for age), cell count/mm3 | Naive CD8+ T cells^a^ (normal range for age), % | CD19+ B cells^a^ (normal range for age), cell count/mm^3^ | CD16+ CD56+ NK cells, 100-700/mm^3^ | AST^a^ (normal value for age), U/L | ALAT, <45 female, <35 male U/L |
| --- | --- | --- | --- | --- | --- | --- | --- | --- |
| **1** | 235 (608-1217) | 53.1 (49.3-72.0) | 84 (228-577) | 86.1 (62.3-86.3) | 24 (119-578) | 70 | 78 (<35) | 68 |
| **2** | U | U | U | U | U | U | 41 (<47) | 66 |
| **3** | 866 (560-1067) | 60.2 (49.4-71.9) | 90 (216-499) | 61.2 (48.6-87.5) | 9 (114-436) | 216 | 18 (<35) | 14 |
| **4** | 394 (608-1217) | 70.1 (49.3-72.0) | 514 (228-577) | 73.8 (62.3-86.3) | 34 (119-578) | 47 | 29 (<35) | 15 |
| **5** | 470 (695-1473) | 70.3 (53.4-74.7) | 457 (426-991) | 68.9 (49.2-82.7) | 210 (116-555) | 317 | 32 (<35) | 14 |
| **6** | 491 (560-1067) | 46.6 (49.4-71.9) | 298 (216-499) | 13.5 (48.6-87.5) | 45 (114-436) | 98 | U | 31 |
| **7** | 32 (560-1067) | 3 (49.4-71.9) | 48 (216-499) | 28 (48.6-87.5) | 0 (114-436) | 13 | 19 (<35) | 15 |
| **8** | 749 (608-1217) | 70 (49.3-72.0) | 582 (228-577) | 83.3 (62.3-86.3) | 140 (119-578) | 70 | 29 (<30) | 26 |
| **9** | 130 (400-1300) | U | 250 (200-700) | U | 10 (100-400) | 170 | 19 (<31) | 17 |
| **10*** | 0 (400-1300) | U | 10 (200-700) | U | 0 (100-400) | 0 | 113 (<35) | 203 |
| **11** | 290 (400-1300) | U | 1130 (200-700) | U | 60 (100-400) | 20 | 23 (<31) | 13 |
| **12*** | 30 (400-1300) | U | 90 (200-700) | U | 0 (100-400) | 40 |  |  |
| **13** | 790 (400-1300) | U | 390 (200-700) | U | 470 (100-400) | 70 (100-400) | U | 33 |
| **14** | 1000 (400-1300) | U | 600 (200-700) | U | 200 (100-400) | 100 | 24 | 16 |
| **15** | U | U | U | U | 63 (100-500) | U | 17 | 18 |
| **16** | 690 (400-1300) | U | 390 (200-900) | U | 230 (100-500) | 250 (90-600) | U | 18 |
| **17** | U | U | U | U | U | U | 21 | 22 |
| **18** | 300 (400-1300) | U | 0 (200-700) | U | 100 (100-400) | 0 | 46 | U |
| **19** | 320 (400-1300) | U | 1150 (200-900) | U | 100 (100-500) | 40 (90-600) | 21 | 11 |
| **20** | 365 (560-1067) | 71.6 (49.4-71.9) | 127 (216-499) | 61.3 (48.6-87.5) | 46 (114-436) | 31 | 26 (<35) | 22 |
| **21** | 256 (560-1067) | 62.4 (49.4-71.9) | 165 (216-499) | 58.3 (48.6-87.5) | 20 (114-436) | 39 | 37 (<35) | 20 |
| **22** | 308 (400-1400) | U | 183 (200-900) | U | 173 (100-500) | 62 | U | 10 |
| **23** | 273 (925-2477) | 66 (71.5-84.2) | 222 (394-1197) | 75 (57.1-91.4) | 23 (686-1732) | 29 | 69 (<47) | 65 |
| **24** | 990 (560-1067) | U | 480 (300-800) | U | 140 (200-500) | 160 | 24 | 25 |
| **25** | 560 (400-1300) | U | 320 (200-700) | U | 270 (100-400) | 190 (100-400) | 18 | 15 |
| **26** | 868 (400-1300) | 66 (49.4-71.9) | 717 (200-700) | 75.5 (49.2-82.7) | 197 (100-400) | 76 | 44 | 38 |
| **27** | 982 (560-1067) | 74.6 (49.4-71.9) | 498 (200-700) | 80.4 (49.2-82.7) | 288 (100-400) | 102 | 39 | 35 |
| **28** | 792 (560-1067) | 74.6 (53.4-74.7) | 200 (200-700) | 20.7 (49.2-82.7) | 316 (200-500) | 100 | 35 | 26 |
| **29** | 660 (560-1067) | U | U | U | U | U | U | U |

*#= Laboratory values were obtained at representative time-points in the disease course*. **= Laboratory values were collected during HLH. U= unknown.*

**Supplementary Table 3A: Residual ADA2 activity in a cohort of 29 DADA2 patients before initiation of TNFi**

| **1** | **2** | **3** | **4** | **5** | **6** | **7** | **8** | **9** | **10** | **11** | **12** | **13** | **14** | **15** | **16** | **17** | **18** | **19** | **20** | **21** | **22** | **23** | **24** | **25** | **26** | **27** | **28** | **29** |
| --- | --- | --- | --- | --- | --- | --- | --- | --- | --- | --- | --- | --- | --- | --- | --- | --- | --- | --- | --- | --- | --- | --- | --- | --- | --- | --- | --- | --- |
|  |  | 2.5 | 0.9 |  | 0.3 | 1.5 | 1.3 |  |  |  | 0.1 |  | 1.4 | 0 | 0.17 |  | 0.4 | 0.3 | 0.2 | 0.6 | 0 | 0.3 | 0 | 0.1 |  |  | 0.1 |  |

**Supplementary Table 3B: Residual ADA2 activity in a cohort of 29 DADA2 patients after start TNFi**

| **1** | **2** | **3** | **4** | **5** | **6** | **7** | **8** | **9** | **10** | **11** | **12** | **13** | **14** | **15** | **16** | **17** | **18** | **19** | **20** | **21** | **22** | **23** | **24** | **25** | **26** | **27** | **28** | **29** |
| --- | --- | --- | --- | --- | --- | --- | --- | --- | --- | --- | --- | --- | --- | --- | --- | --- | --- | --- | --- | --- | --- | --- | --- | --- | --- | --- | --- | --- |
| 0.15 |  | 1.1 | 0 | 0 | 0 |  |  |  |  |  |  |  | 0.13 |  | 0.2 |  | 0.1 |  |  |  |  |  |  |  |  |  |  | 0.23 |

**Supplementary Table 4: Clinical manifestations before and after start TNF-inhibition in a cohort of DADA2 patients (N=29)**

*Column B (Before) shows clinical manifestation before the onset of TNF-inhibition and column A (After) shows clinical manifestations that developed or did not improve under TNF-inhibition.*

|  | **1** | | **3** | | **4** | | **5** | | **6** | | **7** | | **8** | | **9** † | | **11** | | **14** | | **15** | | **16** | | **17** | | **18** | | **20** | | **21**† | | **22*** | | **25** | | **26** | | **27** | | **28** | | |
| --- | --- | --- | --- | --- | --- | --- | --- | --- | --- | --- | --- | --- | --- | --- | --- | --- | --- | --- | --- | --- | --- | --- | --- | --- | --- | --- | --- | --- | --- | --- | --- | --- | --- | --- | --- | --- | --- | --- | --- | --- | --- | --- | --- |
|  | **B** | **A** | **B** | **A** | **B** | **A** | **B** | **A** | **B** | **A** | **B** | **A** | **B** | **A** | **B** | **A** | **B** | **A** | **B** | **A** | **B** | **A** | **B** | **A** | **B** | **A** | **B** | **A** | **B** | **A** | **B** | **A** | **B** | **A** | **B** | **A** | **B** | **A** | **B** | **A** | **B** | | **A** |
| Cutaneous involvement (except eczema) | X |  | X |  | X |  | X |  |  |  | X |  |  |  |  |  |  |  | X |  |  |  | X |  | X |  | X |  | X |  | X |  | X |  | X |  | X |  | X |  | X |  | |
| Eczema | X |  |  |  |  |  |  |  |  |  |  |  |  |  |  |  |  |  |  |  |  |  |  |  |  |  |  |  |  |  | X | X |  | X |  |  |  |  | X | X |  | X | |
| Fever | X |  |  |  | X |  | X |  |  |  |  |  | X |  |  |  |  |  | X |  |  |  |  |  | X |  |  |  |  |  |  |  |  |  | X |  | X |  |  |  |  |  | |
| Stroke | X |  |  | X^1^ |  |  |  |  | X |  | X |  | X |  |  |  |  |  |  |  |  |  |  |  |  |  | X | X^2^ | X |  | X |  | X |  |  |  |  |  | X |  | X |  | |
| PAN-like rash or other cutaneous vasculitis |  |  | X |  |  |  |  |  | X |  | X |  |  |  |  |  |  |  | X |  |  |  | X |  |  |  | X |  | X |  | X |  | X |  |  |  |  |  |  |  | X |  | |
| Arthralgia/  artritis | X |  |  |  | X |  | X |  |  |  |  |  | X | X |  |  |  | X |  |  |  |  |  |  |  |  |  |  |  |  |  |  |  |  | X |  | X |  |  |  |  |  | |
| IBD-like disease |  |  |  |  | X |  |  |  |  |  | X |  |  |  |  |  |  |  |  |  |  |  |  |  |  |  |  |  |  |  |  |  |  |  |  |  |  |  |  |  |  |  | |
| Aphthous stomatitis | X |  |  |  | X |  | X |  | X |  |  |  | X |  | X | X | X | X |  |  |  |  |  |  |  |  |  |  |  |  |  |  |  |  | X |  |  |  | X |  |  |  | |
| Anemia | X |  |  |  |  |  |  |  |  |  | X |  |  |  |  |  | X | X |  |  | X | X |  | X |  |  |  |  |  |  |  | X | X | X |  |  |  |  |  |  |  |  | |
| Thrombocytopenia | X |  |  |  |  |  |  |  |  |  | X | X |  |  | X | X |  |  |  |  |  |  |  |  |  |  | X | X |  |  |  | X |  |  |  |  |  |  |  |  |  |  | |
| Neutropenia | X |  |  |  | X | X |  |  |  |  | X |  |  |  | X | X | X | X |  |  | X | X |  |  |  |  |  |  |  |  | X | X |  |  |  |  |  |  |  |  |  |  | |
| Lymphopenia | X |  |  |  |  |  |  |  |  |  | X | X |  |  | X | X |  |  |  |  | X | X |  |  |  |  | X | X | X | X | X | X |  |  |  |  |  |  |  |  |  |  | |
| Hypogammaglobulinemia | X | X |  |  |  |  |  |  | X | X | X | X | X | X | X | X |  |  | X | X |  |  |  |  |  |  | X | X | X | X | X | X |  |  |  |  | X | X |  |  |  |  | |

*B= before start anti-TNF; A= after start anti-TNF; U= unknown. Predominant vasculopathy phenotype patients are red (n=7), predominant hematologic phenotype patients are blue (n=2), mixed phenotype patients are green (n=12). Mixed phenotype indicates a combination of two or three phenotypes (vasculopathy, hematologic manifestations, immunodeficiency). Patients 10, 23, 24 and 29 were excluded from this table as their response to TNFi could not be reliably assessed. *= Not considered hematologic phenotype as anemia resolved after iron suppletion. X^1^= suspected stroke developed during a period of poor response to TNFi; X^2^= clinical symptoms suspected of stroke without MRI-abnormalities.*
